# Physicochemical, Quality, Antioxidant and Sensory Attributes of Innovative Functional White Pan Bread Manufactured from Wheat Flour Fortified with Broccoli Plant Parts

**DOI:** 10.3390/foods15193426

**Published:** 2026-09-25

**Authors:** Otilia Cristina Murariu, Anca Elena Calistru, Florin Murariu, Petru-Marian Cârlescu, Florina Stoica, Aida Albu, Gabriela Frunză, Nadezhda Golubkina, Gianluca Caruso

**Affiliations:** 1 Department of Food Technologies, ‘Ion Ionescu de la Brad’ Iasi University of Life Sciences, 700490 Iasi, Romania; otilia.murariu@iuls.ro (O.C.M.);; 2 Department of Pedotechnics, “Ion Ionescu de la Brad” Iasi University of Life Sciences, 700490 Iasi, Romania; petru.carlescu@iuls.ro; 3 Department of Agroeconomy, “Ion Ionescu de la Brad” Iasi University of Life Sciences, 700490 Iasi, Romania; 4 Research Institute for Agriculture and Environment, “Ion Ionescu de la Brad” Iasi University of Life Sciences, 700490 Iasi, Romania; 5 Federal Scientific Vegetable Center, 143072 Moscow, Russia; 6 Department of Agricultural Sciences, University of Naples Federico II, Via Università 100, 80055 Portici, Naples, Italy; gcaruso@unina.it

**Keywords:** *Brassica oleracea* L. var. italica Plenck, innovative bakery products, physicochemical properties, porosity, color, vitamin C, polyphenols, carotenoids, antioxidant activity

## Abstract

Innovative aliments obtained upon integration of plant-based ingredients for producing nutraceutical-enriched functional food under the perspective of sustainable management have been drawing increasing importance within the scientific community. Research was conducted to evaluate the effects of the addition of broccoli plant part powder (stalks and inflorescences) at different concentrations (3%, 5% and 10%) into dough on textural, porosity, quality, antioxidant and sensory characteristics of ‘White Pan Bread’. The rising addition (from 3 to 10%) of broccoli stalks and inflorescences into ‘White Pan Bread’ led to a gradual decrease in most textural properties and color components of dough, crumb and crust, as well as dry matter and water activity. Contrary, mineral substances, pH, acidity, and the antioxidant compounds and activities showed increasing trend. Among the sensory features, the scores of general bread aspect as well as crumb texture, structure, aeration, sweetness and aroma aspects decreased with the increasing broccoli powder integration. The latter augmentation fostered crust and crumb flexibility, freshness, sulfur, cruciferous and plant notes. Overall, manufacturing broccoli-enriched bread allows to create a novel functional aliment with less crumb softness and color intensity, importantly characterized by enhanced mineral and bioactive properties within the sustainability framework.

## 1. Introduction

Lately, the bakery industry has experienced significant growth in the development of innovative products meeting consumers’ expectations for health, sustainability, and personalized nutrition [1]. Bread is among the most important and widely consumed baked aliments, produced through grain processing and characterized by affordability, high energy content, and widespread acceptance among consumers as a staple food, with Europe having a very long tradition and great diversity [2]. In Romania, this product has an important role in population diet, which gives it a strategic focus in nutritional implementation and development of functional foods upon addition of plant ingredients rich in bioactive compounds [3,4]. Particularly, vegetables, fruits, legumes, nuts and tea show high levels of antioxidants, like polyphenols, and antioxidant activity, and improve the final product’s shelf-life [5].

Innovative functional aliments obtained by adding waste or beneficial substances [6,7] are defined as “natural or processed foods that contain effective and non-toxic amounts of bioactive compounds, which provide clinically proven health benefits documented through the use of specific biomarkers for the prevention of human aging, the treatment of chronic diseases, or their symptoms” [8].

Previous investigations have reported the incorporation of plant-based ingredients or byproducts to develop functional foods [9], particularly bread rich in dietary fiber, vitamins, and phytochemicals [3].

Current trends in the bakery sector are aimed at simultaneously improving nutritional profiles and valorizing food industry byproducts thus reducing environmental impact. In this respect, bread provides complex carbohydrates, plant-based protein, B vitamins, and essential minerals and the moderate consumption of this product, particularly made from whole-grain flours or enriched with functional ingredients, can contribute to dietary fiber intake, maintain gut health, and reduce the risk of metabolic disorders [2]. The acute health effects of bread include appetite suppression, reduced diastolic blood pressure, improvements in glycemia, insulinemia and satiety [10].

Broccoli (*Brassica oleracea* L. var. italica Plenck), a species belonging to the *Brassicaceae* family, is among the most promising sources of functional ingredients for bakery products because of its high levels of dietary fiber, vitamins (A, C, E, and K), minerals, phenolic compounds, carotenoids, and glucosinolates, i.e., bioactive compounds providing antioxidant, anti-inflammatory, and chemoprotective effects [4,11].

According to FAO [12], world production of broccoli and cauliflower overcame 26 million tons in 2023, reflecting growing consumer interest in these vegetables due to their health benefits [11], predominantly produced in China, India, Spain, Italy, United States and Mexico [12]. Considering that the edible florets account for about 15% of the plant total biomass, processing broccoli generates significant amounts of waste represented by leaves, stalks, and roots, frequently discarded as agricultural or industrial waste, with stalks representing about 21% of total biomass, i.e., 1.4 times the edible inflorescence yield [11]. The mentioned byproducts contain significant concentrations of dietary fiber, polyphenols, flavonoids, and glucosinolates, offering high potential for food utilization [11].

In the current context of the circular economy and European strategies to reduce food waste and environmental impact, transforming broccoli byproducts into innovative functional ingredients represents a sustainable solution for recovering bioactive compounds and reintegrating them into the food chain [11].

Although most existing literature confirms the potential of *Brassica oleracea* var. italica and its byproducts as functional ingredients for bakery products [13], it is focused only on broccoli florets [4] or single tissue powder [14]. Innovatively, the present research simultaneously compared the effects of broccoli stalk and inflorescences, at three addition concentrations, on the textural, quality, antioxidant and sensory characteristics of bread, within the concept of circular economy.

The plant part inclusion relates to the contribution in dietary fiber, which influences rheological and technological properties of dough [15], in antioxidant compounds and overall sensory attributes [16].

Considering that conventional bakery aliments, made exclusively from refined white flour, have limited bioactive compound content, the main target of this research consisted in improving bread nutritional properties by adding broccoli (*Brassica oleracea* L. var. italica) stalk and inflorescence powder, at 3, 5 and 10% concentrations, hypothesizing that the mentioned experimental treatments would affect the textural, porosity, quality, color, antioxidant and sensory characteristics of these innovative products.

## 2. Materials and Methods

### 2.1. Research Location and Experimental Protocol

Research was carried out at the Department of Food Technologies (Iasi University of Life Sciences, Romania), in 2026, with the purpose of evaluating the effects of the addition of broccoli (*Brassica oleracea* L. var. italica Plenck) stalk and inflorescence powder (Table 1) into ‘White Pan Bread’ on the textural, quality, antioxidant and sensory characteristics of the final product. The chemical composition of broccoli plant part powder (Table 1) was determined according to the analytical methods reported in the next sub-sections, with three replicates per each measurement.

Six treatments obtained by combining three addition percentages (3, 5 and 10%) of two broccoli plant parts (stalks and inflorescences) into ‘White Pan Bread’, plus an untreated control, were compared using a completely randomized design with three replicates; the latter correspond to three independent production batches (each including three bread loaves) associated with each formulation. The latter procedure allowed us to make all the possible comparisons between the six treatments and between each of them with the untreated control. Indeed, we observed and described the trends of each variable as a function of the addition percentage within each broccoli plant part and the comparison between the two plant parts at each integration percentage. The untreated control, containing the same basic ingredients as the experimental treatments except for broccoli additions, is essential to assessing the differences arising from *B. oleracea* var. italica stalks and inflorescences incorporation.

### 2.2. Preparation of the Raw and Auxiliary Materials

Fresh broccoli (*Brassica oleracea* L. var. italica) at commercial maturity were purchased at a local supermarket in Iași, Romania. All broccoli samples were obtained from the same commercial batch to minimize raw material variability. Prior to processing, the broccoli heads were visually inspected, and only healthy stalks and inflorescences free from mechanical damage or visible defects were selected.

The stages of the technological process for drying broccoli began with the receiving and sorting of the raw material (Figure 1). The fresh broccoli were received and subjected to rigorous sorting to remove damaged parts, mechanically damaged tissue, or plant debris. The plant raw material was washed with drinkable water to remove adhering impurities and reduce the surface microbial load, after which the florets were separated from the stalks, thus obtaining two distinct plant fractions.

The plant components were processed using a Philips^®^ (Series 7000, Amsterdam, Holland) food processor until a homogeneous plant mass was obtained (Figure 2a,b), with small particle sizes conducive to mass transfer during drying. The chopped plant material was evenly distributed in a single uniform thin layer on dehydrator stainless-steel mesh trays to ensure homogeneous air circulation during drying. The mentioned process was performed in a forced-air food dehydrator (GGMGastro, Ochtrup, Germany) at 45 °C for 24 h. The latter stage allows for controlled moisture reduction, limiting both heat-labile bioactive compound and natural pigment degradation. After dehydration, the material was ground using a Moulinex laboratory grinder (Moulinex, Écully, France) until a fine powder was obtained and subsequently sieved obtaining a homogeneous particle dimension fraction (Figure 2c,d). The broccoli plant part powder was immediately transferred into airtight, light-protected containers and stored under dry conditions at room temperature until analyses.

Standardized formulation and technology were followed to obtain the control sample, blending white flour, water, fresh yeast, salt and sugar, as presented in Table 2 for the untreated and broccoli-enriched bread recipes. The experimental formulations were prepared by partially replacing wheat flour with broccoli powder at levels of 3%, 5%, and 10% (*w*/*w*). The water addition was individually adjusted for each formulation to achieve comparable dough consistency and handling characteristics. The adjustment was performed based on the hydration behavior and compositional characteristics of broccoli powder, with the aim of obtaining doughs with adequate hydration, elasticity, and a well-developed gluten network, and was determined by preliminary baking trials. Indeed, since broccoli powder has a higher content of dietary fiber than wheat flour, its incorporation increases the water absorption capacity of the flour blend. Consequently, the slight increases in water addition with the rising percentage of broccoli powder up to 10% compensate for the higher water-binding level of the fiber-rich material and to maintain comparable dough rheological characteristics. This ensured technological consistency and comparable processing behavior among all experimental samples.

Premium quality white wheat flour Băneasa type 000 (M.P. Băneasa Moara S.A., Buftea, Romania) was used in this research, with the following contents of the main chemical components reported by the manufacturer: 14.0% moisture, 12.5% proteins, 0.5% ash, 26% wet gluten content.

The yeast was stored in a 700 L refrigerator at 0–4 °C until sample preparation, after which the flour and broccoli powder were stored at 20 °C.

Preparation of raw materials involved the following steps: blending the flours to obtain a homogeneous batch in terms of baking properties, in order to ensure a stable production process and consistent quality of the finished product. Water preparation consisted of bringing the water to the temperature required to achieve the desired thermal parameters for the dough, and mixing cold tap water with water heated to approximately 60 °C. Yeast was prepared as a suspension using a portion of the water intended for dough preparation, heated to 30–35 °C, with the purpose to ensure its uniform distribution throughout the dough mass. The salt was dissolved in water to obtain a saturated solution, which was filtered to remove impurities.

### 2.3. Bread Preparation

In this study, ‘White Pan Bread’ was produced using a conventional lean wheat bread formulation consisting of wheat flour, water, compressed baker yeast and salt. The dough was placed into rectangular baking tins, proofed under controlled conditions and baked to obtain pan bread loaves.

The technological flow related to ‘White Pan Bread’ produced from *Triticum aestivum* flour (000 type), by the dough direct preparation method, is shown in Figure 3 and Figure 4, in compliance with the ISO 6820-1985 ‘Wheat flour and rye flour—General guidance on the development of bread-making tests’ [17]. In this respect, seven batches of bread (with 3 loaves each), corresponding to six formulations (B) with broccoli additions plus an untreated control, with three loaves per batch, were manufactured under the same conditions.

Dough kneading is the process consisting of two successive stages: mixing phase and kneading itself. During the mixing phase, using a 7/SN Fimar spiral mixer^®^ (Fimar, Sofia, Bulgaria) with 7 kg production capacity, the components were thoroughly mixed and hydrated. Particularly, the flour particles absorb water, swell, and form small moist agglomerates, and the duration depends on the flour particle size and temperature, as colder or finer flours require longer times. In the present research, the dough was kneaded for 4–5 min in the first stage, followed by 2–3 min in the second stage. During the actual kneading phase, the formed clumps joined and homogenized under enzymatic action, thus forming the dough gluten network and internal structure, which provide stretchability and elasticity. Resting/fermentation took place immediately after the completion of the kneading process and lasted for 1 h at 32 °C and 85% relative humidity. The mentioned process extended from the end of mechanical homogenization of dough until the latter was directed toward the structural division phase. After bulk fermentation, the dough was split into 850 g portions using a semi-automatic hydraulic dough divider (I20S). A pre-shaping was applied to optimize the bread porous texture and, at this stage, the pores which had been opened by division are closed, resulting in uniform, fine, and regular porosity. Then, the divided dough pieces were manually shaped into loaves, placed in baking pans, subjected to final proofing, and baked. The intermediate rest, allowing to relieve the internal stresses generated during division and pre-shaping, lasted 4–5 min in non-air-conditioned spaces. Next, the dough was divided into 3 equal portions (850 g each) immediately subjected to textural determinations and, to prevent their fermentation, each of them was divided into 2 portions: one half was split into 3 parts of 50 g and modeled as 100 × 100 mm spheres, then spread on the flat creating a 5 mm thickness; the second half was modeled as spheres spread into a flat form and then divided in strips, 100 mm long, 15 mm wide and 5 mm thick.

The 3 portions of each experimental treatment were shaped into the final dough, which was formed into a tray loaf. Shaping is the stage of creating ’White Pan Bread’-style loaves from the dough pieces, with the aim of establishing their structure to ensure uniform development during proofing and baking. Controlled surface tension contributed to the volume and shape stability of the bread. Final fermentation/proofing took place in a batch process, in a proofing chamber at 31 °C and 87% relative humidity for 55 min, under controlled climate conditions. Water sprinkling is the process of moistening the surface of the dough with water before baking, to delay crust formation, promote oven spring, and impart a glossy finish and thin crust without uncontrolled cracks. Baking is the stage in which the dough is transformed into bread through the application of thermal energy in bakery ovens. The latter conversion results from a series of physicochemical processes occurring within the dough as it heats up, whose nature and intensity are determined by the applied thermal regime. Baking was performed at 200 °C for 50 min in an electrical rotary convection oven (Zucchelli Forni S.p.a., Trevenzuolo, Verona, Italy). In the first 15 min, crust formation was prevented by using steam, thus avoiding blocking the sample expansion in the oven. Cooling takes place in the first few hours after removal from the oven and its duration depends on product mass and shape, as well as the air parameters in the storage area. In this research, the bread loaves were cooled at 20 ± 2 °C for approximately 3 h, stored at 18–20 °C and 65–70% relative humidity for 4 h, then kept in polyethylene film to preserve freshness, at 20 °C for 24 h and at 2–4 °C for the next 9 days.

Physicochemical, quality, color, and antioxidant analyses were performed within 24 h after baking. Sensory evaluation was conducted on fresh bread samples in the same day after cooling, to ensure uniform assessment conditions and to minimize changes associated with storage.

### 2.4. Determination of Bread Textural Properties and Porosity

The texture of the experimental samples was measured with the Mark 10 Plus texturometer (Mark-10 Corporation, Copiague, NY, USA), as previously reported [18]. The following parameters were measured on dough: Maximum breaking strength (MBS), maximum deformation (MD), mechanical work (MW), firmness, consistency, cohesiveness, elasticity, resilience, gumminess, and chewiness.

Two sample geometries were employed to obtain complementary information on dough mechanical behavior. Spherical samples were used for compression tests to evaluate dough firmness and compressibility under localized deformation, whereas strip-shaped samples were subjected to tensile tests to determine dough extensibility and resistance to extension, reflecting the development and integrity of the gluten network. The combined use of compression and tensile testing provided a more comprehensive assessment of dough rheological properties and its technological performance during processing.

Open, closed, and total porosity, were determined using the Computed Tomography system (micro-CT SkyScan 1273, Bruker, Billerica, MA, USA) and expressed in %, as previously reported [19].

### 2.5. Determination of Quality Parameters, Water Activity and Color Components of Bread

The dry matter (%) of bread crumb was measured upon drying 5 g of each sample at 130 °C for 1 h, whereas the pH and titratable acidity were determined in each 25 g bread loaf, according to AOAC International [20].

Proteins were analyzed using the Kjeldahl method and lipids by the Soxlet method, in compliance with AOAC International [20]; the latter reference was also used for sugar and fiber determinations. Mineral substances (ash) were measured as residues of sample calcination at 550 °C for 6 h and NaCl content upon chlorine ion titration with AgNO_3_ in the presence of K_2_CrO_4_ as an indicator [20]. Briefly, a known mass of bread sample was homogenized in distilled water, filtered, and an aliquot was titrated. The NaCl content (%) was calculated considering the titration volume, AgNO_3_ normality, dilution factor, and sample mass, and expressed on a fresh weight basis. All measurements were performed in triplicate. To ensure data accuracy, moisture correction and mass-balance verification were performed by comparing experimentally determined NaCl values with theoretical values derived from the formulation. The analytical recovery was within acceptable limits (90–110%), confirming the reliability of this method.

Water activity of bread samples was assessed by a humimeter RH2 (Schaller Messtechnik GmbH, Sankt Ruprecht an der Raab, Austria), as previously reported [16].

The color components (CIE Lab system, L*, a*, b*) of bread were measured in 3 replicates by 400 trichromatric reflectance Minolta Colorimeter (Konica Minolta Sensing INC., Osaka, Japan) [1].

### 2.6. Extraction of Bioactive Compounds

Bioactive compounds were extracted from broccoli powder and bread samples using ultrasound-assisted solvent extraction. Briefly, 1 g of dried broccoli powder or bread crumb was homogenized with 10 mL of 70% (*v*/*v*) ethanol for the extraction of phenolic compounds and separately with 10 mL of a hexane:acetone (3:1, *v*/*v*) mixture for carotenoid extraction. The suspensions were homogenized at room temperature for 5–6 min and subsequently subjected to ultrasound-assisted extraction in an ultrasonic bath at 40 kHz and 40 °C for 45 min. The extracts were centrifuged at 5000 rpm for 10 min at 4 °C, and the resulting supernatants were collected for measuring carotenoids, polyphenols, flavonoids and antioxidant activity.

### 2.7. Determination of Antioxidant Activity

The antioxidant activity of the extracts was measured using the DPPH (2,2-diphenyl-1-picrylhydrazyl) radical scavenging assay [21], quantified by a Trolox standard calibration curve and expressed as µmol Trolox equivalents (TE) g^−1^ dry weight.

### 2.8. Determination of Vitamin C

The vitamin C content was determined by 2.6-dichlorophenolindophenol (DCPIP) titrimetric method [22]. The sample was extracted with 2% (*w*/*v*) oxalic acid to minimize the oxidation of ascorbic acid during sample preparation. The extract obtained was titrated with a standardized 2,6-dichlorophenolindophenol (DCPIP) solution until the appearance of a persistent pale pink color lasting approximately 15 s, which was considered the endpoint of the titration. Vitamin C content was calculated using the standardization factor of the DCPIP solution obtained with L-ascorbic acid and expressed as mg ascorbic acid (AA) 100 g^−1^ fresh weight.

### 2.9. Determination of Total Polyphenol Content

Total polyphenol content was determined by the Folin–Ciocâlteu colorimetric method [23], measuring the absorbance by a UV-Vis Specord 210 Plus spectrophotometer (Analytik Jena, Jena, Germany) at 765 nm against a reagent blank, using gallic acid (GA) as calibration curve standard, and was expressed as mg gallic acid equivalents (GAE) g^−1^ dry weight.

### 2.10. Determination of Flavonoid Content

The total flavonoid content (TFC) was determined by the aluminum chloride colorimetric method [24], measuring the absorbance at 510 nm against a reagent blank, using a catechin calibration curve, and was expressed as mg catechin equivalents (CE) g^−1^ dry weight.

### 2.11. Determination of Total Carotenoids, β-Carotene and Lycopene

Total carotenoids, β-carotene and lycopene were measured as previously described [25].

### 2.12. Sensory Evaluation of Bread Products

The sensory characteristics of the bread samples corresponding to the 7 experimental treatments applied were determined by QDA (Quantitative Descriptive Analysis) as previously reported [19], at “Ion Ionescu de la Brad” University for Life Sciences in Iasi (Romania). A questionnaire was applied to a panel made of 40 people (20 women and 20 men) with experience in the field, aged between 25 and 50, non-smokers, without known cases of food allergies, to obtain a detailed sensory profile of innovative bread products. This study was reviewed and approved by the Ethics Committee for Research in Human Beings of the Department of Food Technologies, Faculty of Agriculture, of the mentioned University, and managed in compliance with the European Union Guidelines of Ethics and Food-Related Research [26]. The bread samples (2 cm thick and 25 g weighed slices) were randomly placed on platters [27] and selected visual and texture-related characteristics of the bread samples were assessed on a 0 to 2 scale: general aspect, crumb texture and structure, aeration, humidity/stickiness, volume and color, crust thickness and color, elasticity, spreadability, and flexibility. The scale was designed to characterize the degree to which the evaluated bread attributes corresponded to the predefined quality descriptors associated with each score. A 1 to 10 numerical intensity scale, anchored at 0 = not perceived/absent and 10 = extremely intense, was used to evaluate the perceived intensity of the following aroma and taste-related sensory descriptors independently: the main aroma (smell + taste) sensory features, such as salt and sweet, overall aroma, sulfur, cereal, fresh bread, aroma acceptability, freshness, cruciferous, nut, yeast, mineral, plant notes, grassy, burned, bitter and caramelized.

The intensity scores were used exclusively to characterize the sensory profile of each formulation and were not derived from, or converted to, the 0–2 compliance scores. The 0–2 and 1–10 scales served different and complementary analytical purposes: the 0–2 scale assessed conformity with predefined bread-quality descriptors, the 1–10 scale quantified the perceived intensity of specific sensory attributes. No transformations or conversions were performed.

### 2.13. Statistical Analysis

Data were processed by analysis of variance (ANOVA) and the mean separations were performed through the Duncan’s test, with reference to *p* < 0.05, in the cases of statistical significance of a measured variable, to simultaneously compare the experimental treatments between each other and with the untreated control. Correlations were performed by Pearson correlation coefficient. SPSS software version 30 was used for data statistical processing.

## 3. Results and Discussion

### 3.1. Textural, Porosity, Quality and Color Characteristics of Dough and Bread

The textural parameters, measured both on flat spherical and strips dough, decreased with the rising addition of broccoli powder, derived from both stalks and inflorescences (Table 3).

Broccoli incorporation led to a progressive reduction in dough strength and extensibility, an effect dependent on both the plant part and the percentage added, with the untreated control showing the highest values of MBS, MDDS, and MW (Table 3). Compared to the control, the 10% addition led to the maximum breaking strength decrease by 55.8% and the deformation energy reduction by 71.7%, on average, that indicates a reduced ability of the dough to resist point stretching, which may foreshadow lower stability during proofing and reduced gas retention capacity [28]. MBS decreased by 73.3% and 69.4% in the dough upon the 10% addition of broccoli stalks and inflorescences, respectively, compared to the control.

The mentioned decrease in the textural parameter values upon the incorporation of plant powders into bakery products suggests a weakening of the viscoelastic matrix due to the increase in insoluble fiber content which frequently reduces porosity, elasticity and volume [9]; the influence on the latter parameter was presumably due to the activity of cruciferous plant enzymes which are inactivated by thermal pretreatment [14]. Other authors [29] reported an appreciable leavening degree of wheat bread integrated with tomato, beetroot and carrot.

In previous research [4,11], the incorporation into bread of 4% broccoli powder rich in fiber and bioactive compounds modified the bread textural characteristics. In this respect, broccoli insoluble fibers have a high capacity for binding water through hydrogen bonds, competing directly with wheat proteins for available water in the dough and, without sufficient free water, gliadin and glutenin molecules cannot unfold or move freely, limiting their transition to a continuous viscoelastic network [30]; moreover, their particles insert themselves between protein chains, disrupting the structural continuity of gluten. The weakening and fragmentation of the viscoelastic gluten film caused by insoluble fibers creates thin and brittle dough cell walls; coarse fiber particles mechanically puncture the expanding gas bubbles during fermentation, which coalesce upon cell wall rupture and CO_2_ escapes prematurely from the dough, resulting in structural collapse and reduced final specific volume, i.e., dense and compact product [31].

Pearson correlations showed negative relationships between the percentage of addition and the textural parameters of spherical dough (r = −0.949 for MBS, r = −0.996 for MDDS, and r = −0.971 for MW) and strips dough (r = −0.875 for MBS, r = −0.938 for MDDS, and r = −0.995 for MW). These values confirm that the increased integration percentage is mainly responsible for the loss of extensibility and the mechanical work required to deform the dough.

The inflorescences had a stronger effect than stalks on MBS and MW in the spherical dough, at 3% addition (25.3% and 10.1% reduction, respectively, compared to the control) and at 10%. The latter difference is presumably due to a more complex composition of the inflorescence, rich in fine plant particles and bioactive compounds that can interfere more intensely with the hydration of gluten proteins, which depends on the nature, particle size, hydration capacity and integration percentage of the added ingredient [32].

Overall, the data demonstrate that the additions of 3–5% are more technologically balanced, as textural parameter reduction remains moderate, particularly for stalk powder. Contrastingly, the 10% level drastically affected the dough strength and extensibility, suggesting an increased risk of obtaining a denser crumb with reduced specific volume and porosity. From an application perspective, stalk powder appeared more favorable for the formulation of functional bread, while inflorescence powder, although potentially richer in bioactive compounds, would require optimization of hydration, kneading time, or the use of improvers to compensate for gluten network weakening [13].

Firmness, consistency, cohesiveness, gumminess and chewiness increased from the untreated control to the highest incorporation of broccoli plant part powder, with the inflorescences having a greater impact than stalks except for cohesiveness which was not significantly influenced by plant part (Table 4); elasticity and resilience showed opposite reaction to integration rising in *B. oleracea* var. italica material.

The untreated control exhibited the highest total and open porosity of bread (Figure 5), characterized by a highly interconnected open-cell structure (61.3%) and negligible closed porosity (0.34%), with a decreasing trend of up to 10% integration which showed the lowest value with the stalk powder; an opposite trend was recorded for closed porosity. Other authors reported that crumb porosity and specific volume of bread added with 4% broccoli floret powder increased by 8.8% and 7.3%, respectively, compared to the untreated control [4]. The specific volume reduction is presumably caused by a dilution of gluten after integration of broccoli powder competing for water with starch, thus reducing the amount of fully hydrated starch granules [28].

In previous research [33], the addition of 2% grape marc to pastries provided the best effects on the product elasticity, presumably due to gluten network decrease and fiber augmentation [27].

The creation of a three-dimensional viscoelastic structure able to retain gases, with polymeric proteins as the main component, is triggered by the even distribution of ingredients at the initial mixing, with the breakdown of the spherical proteins and flour hydration [34]. Temperature rise elicits starch granule swelling after water absorption in the medium, and the increase in amylose chain viscosity is the consequence of their leaching out into aqueous intergranular phase; after that, a viscosity decrease is caused by particle physical degradation due to mechanical shear stress and temperature reduction [34].

### 3.2. Quality and Color Characteristics of Dough and Bread with Broccoli Plant Parts

Dry matter and water activity decreased with the rising addition of broccoli powder, derived from both stalks and inflorescences (Table 5), as well as the color component L of dough, crumb and crust, the component a of dough and crust, the component b of crust (Table 6). Contrary, acidity, pH and mineral substances (Table 5), and the component a of bread, the component b of dough and bread crumb showed increasing trend (Table 6).

Bread quality parameters were significantly influenced by the incorporation of broccoli powder, both as plant part and addition percentage. The progressive reduction in the dry matter content with the increasing powder integration suggests a higher water retention capacity in fortified products, which is frequently associated with dietary fibers and hydrophilic polysaccharides in the plant matrix. Compared to the control, the lowest values were recorded under the broccoli inflorescence concentration of 10%, suggesting a higher crumb hydration also due to the highest water supply corresponding to this experimental treatment during dough preparation. This behavior was also reported in recent studies on bakery products enriched with plant fibers, where the increase in water binding capacity led to a decrease in dry matter and texture modification of the final product [35].

Acidity significantly increased with the fortification degree, by 2.8-fold upon 10% broccoli inflorescence incorporation, compared to the control, which suggests a more important contribution of organic acids and bioactive compounds present in this plant part. The acidity increase can have positive implications on bread aromatic profile, but excessive values can affect the sensory acceptability. In parallel, the pH showed moderate variations, which suggests the existence of a buffer effect exerted by proteins and mineral salts in the cereal products. Similar trends were reported for breads fortified with broccoli powder, kale or other plant sources rich in fiber and phenolic compounds, where titratable acidity increased concomitantly with plant material addition [30].

The content of proteins and lipids in bread decreased from the untreated control to the highest broccoli powder addition percentage, both for stalks and inflorescences (Table 5), whereas dietary fiber increased with the rising integration of broccoli ingredients.

Other authors reported that 4% [4] and 5% [36] broccoli powder addition to bread increased dietary fiber content, compared to the control.

In previous research [27,33], the rising integration of grape pomace into pastries caused a significant enhancement of nutritional level, and a reduction in moisture which was increased by fruit incorporation [37].

The addition of broccoli plant part powder into bread did not significantly affect the NaCl content. Water activity (AW) showed a gradual significant decrease from the control to the highest broccoli addition percentage, suggesting a reduction in free water available for microbial growth, allowing the extension of shelf life and the slowing down of decay processes [16].

Bread color parameters were significantly influenced by broccoli powder addition, both as stalks and inflorescences, and the processing stage (dough, crust, crumb). L* values significantly decreased with rising broccoli addition in all processing stages, with reductions of 23.1% in the dough, 26.1% in the crust and 22.9% in the crumb, under the 10% broccoli inflorescence powder addition, compared to the control.

Crust exhibited the lowest lightness values, a phenomenon explained by the high intensity of non-enzymatic browning reactions on the product [38]. In contrast, crumb remained considerably lighter, suggesting less thermal degradation and better preservation of the starch-gluten structure, consistently with recent studies on bread enriched with chlorophyll-rich plant powders, where the thermal gradient between the surface and the core caused major differences in color stability [39].

The bread added with broccoli inflorescence powder exhibited lower L* values, compared to the stalks, suggesting a greater pigmentation capacity, explainable by higher chlorophyll, carotenoid, and phenolic concentration in floral structures. Indeed, the inflorescence produced a more intense visual appearance, closer to functional plant-based products with a “green-enriched” profile, as described in baked goods fortified with kale [40]. Contrastingly, the stalk powder produced more moderate color changes, which may be advantageous in terms of consumer acceptability, as they better preserve the traditional appearance of bread.

The color component a* showed the most significant chromatic change, with values becoming progressively more negative with increasing broccoli material addition. In the dough, the a* value decreased from the untreated control to 10% inflorescence addition percentage, which suggests the development of an intense green hue reflecting the high intake of chlorophyll and chlorophyll-derivatives conveyed by broccoli. In bread crumb, the negative a* values, less pronounced compared to the dough, indicate an accentuation of yellow-green tones due to carotenoids and intermediate products of partial thermal degradation of green pigments during baking heat treatment. Similar results were reported in baked aliments fortified with chlorophyll-rich plant powders, where heat treatment partially converted chlorophylls into pheophytins, leading to olive-green or brown-green hues [39].

Increasing values of the color component b* were recorded from the untreated control to the highest fortification level of 10%, both in the dough and in the crumb, indicating an intensification of yellow hues which can be attributed to the broccoli natural carotenoids, as well as the formation of browning compounds generated by thermal reactions. The color component b* of the crust showed opposite trend, suggesting the predominance of Maillard reactions and the formation of brown pigments on bread surface, presumably due to the balance alteration between yellow and brown tones caused by the plant ingredient integration [38].

The mentioned outcomes suggest a close relationship between broccoli powder concentration and the visual product changes, i.e., the increase in broccoli plant part addition correlated negatively with L* and positively with the intensity of green and yellow hues. These color changes are important either from a technological perspective or from a commercial one, as color is one of the main indicators of consumer perception regarding the product functional and natural character. In this respect, the 3 to 5% broccoli powder integration into bread provided moderate and sensorially acceptable coloring, maintaining the conventional bread visual identity, whereas a 10% addition, particularly from inflorescence powder, produced major color changes, consistently with previous recent studies on bread fortified with plant-based ingredients rich in natural pigments [9]. In other research [4,11], broccoli powder addition to bread led to light yellow with a darkish shade crumb color.

### 3.3. Antioxidant Compounds and Activity of Bread Added with Broccoli Plant Parts

Fortifying bread with rising addition from 3 to 10% of both broccoli stalk and inflorescence powder significantly increased the antioxidant compounds analyzed (Table 7), i.e., vitamin C, total polyphenols (TP), flavonoids, total carotenoids (TC), β-carotene, and lycopene, and antioxidant activity (Figure 6), compared to the untreated control.

The highest vitamin C, total polyphenols (seven-fold increase) and flavonoid contents were recorded under the 10% integration of inflorescences which proved to be a better source of these antioxidants, compared to the stalks. Recent literature on bakery products fortified with kale [40] confirmed that the starch-gluten matrix can exert a partial protective effect on ascorbic acid, limiting oxidative degradation during baking. Broccoli phenolic compounds interact with gluten proteins through hydrogen bonds and hydrophobic relations which block the protein reactive sites, reducing the formation of covalent crosslinks [41].

Similarly to the mentioned antioxidants, total carotenoids, β-carotene and lycopene also showed significant increases, compared to the control, up to 10% broccoli inflorescence added bread, which is important because carotenoids exert both antioxidant role and biological functions associated with cellular protection and the reduction in oxidative stress. Previous studies regarding bread fortified with plant powders rich in natural pigments [42], reported the good thermal stability of these compounds in the cereal matrix after baking.

The antioxidant activities increased from the untreated control to the highest broccoli powder incorporation, either as stalks or inflorescences (Figure 6). However, the inflorescences had a greater impact as also recorded for all the antioxidant compounds examined, compared to the stalks, though both plant parts can be utilized technologically depending on the intended nutritional objective. In other investigations, increases in antioxidant profile and nutritional value, compared to the control, were recorded upon 4% [4] and 5% [36] broccoli powder integration into bread.

According to previous reports [41], the antioxidant activities in foods are affected by polyphenols bound to matrix components and the integrated materials rich in the mentioned bioactive compounds can enhance the bioavailability of flour-based substances; the fortification efficiency is reduced by phenolics–proteins interactions in aliments.

### 3.4. Sensory Features of Bread Added with Broccoli Plant Parts

All the sensory characteristics analyzed, except for overall aroma and aroma acceptability, were significantly affected by the experimental treatments (Table 8 and Table 9).

**Table 8:** Statistically significant differences between the experimental treatments in terms of the sensory features of bread added with broccoli plant part powder.

ET	GA	CTS	CT	CsC	E	Sp	F	CmC	CA	CHS	CV	Su	F
UC	1.86 ± 0.30 a	1.86 ± 0.32 a	1.86 ± 0.30 a	1.43 ± 0.72 c	1.71 ± 0.65 a	1.57 ± 0.67 a	1.43 ± 0.73 c	0.71 ± 0.66 c	1.86 ± 0.32 a	1.86 ± 0.36 a	2.00 ± 0.00 a	0.86 ± 0.36 c	0.86 ± 0.38 d
BBSP3	1.71 ± 0.42 ab	1.71 ± 0.40 ab	1.71 ± 0.41 ab	1.57 ± 0.47 bc	1.71 ± 0.42 a	1.57 ± 0.71 a	1.43 ± 0.51 c	0.71 ± 0.42 c	1.29 ± 0.44 ab	1.71 ± 0.44 ab	1.57 ± 0.46 ab	1.86 ± 1.41 c	3.86 ± 2.03 c
BBSP5	1.57 ± 0.47 bc	1.57 ± 0.45 bc	1.57 ± 0.69 bc	1.71 ± 0.42 ab	1.71 ± 0.42 a	1.43 ± 0.71 ab	1.57 ± 0.46 bc	1.00 ± 0.51 c	1.29 ± 0.44 ab	1.43 ± 0.49 ac	1.29 ± 0.42 bc	2.86 ± 2.42 bc	5.86 ± 2.59 ab
BBSP10	1.43 ± 0.46 c	1.43 ± 0.68 c	1.43 ± 0.46 cd	1.86 ± 0.30 a	1.43 ± 0.68 bc	1.29 ± 0.42 b	1.86 ± 0.32 a	1.57 ± 0.53 ab	0.86 ± 0.79 bd	1.00 ± 0.88 bd	1.14 ± 0.78 bd	3.29 ± 2.90 bc	6.71 ± 2.64 a
BBIP3	1.71 ± 0.41 ab	1.71 ± 0.40 ab	1.71 ± 0.68 ab	1.43 ± 0.45 c	1.57 ± 0.71 ab	1.29 ± 0.42 b	1.43 ± 0.79 c	1.14 ± 0.41 bc	1.14 ± 0.38 bc	1.43 ± 0.49 ac	1.14 ± 0.69 bd	3.00 ± 2.09 bc	4.71 ± 2.55 bc
BBIP5	1.57 ± 0.46 bc	1.57 ± 0.44 bc	1.57 ± 0.46 bc	1.57 ± 0.45 bc	1.29 ± 0.68 cd	1.00 ± 0.73 c	1.57 ± 0.69 bc	1.71 ± 0.44 a	0.57 ± 0.70 cd	0.86 ± 0.90 cd	0.86 ± 0.77 cd	5.14 ± 3.20 ab	5.86 ± 2.67 ab
BBIP10	1.43 ± 0.46 c	1.43 ± 0.47 c	1.29 ± 0.70 d	1.86 ± 0.30 a	1.14 ± 0.82 d	1.00 ± 0.67 c	1.71 ± 0.41 ab	2.00 ± 0.00 a	0.43 ± 0.46 d	0.43 ± 0.68 d	0.57 ± 0.68 d	6.14 ± 3.75 a	6.29 ± 2.69 a

ET: Experimental treatment; UC: untreated control; BBSP3—bread with 3% broccoli stalk powder addition; BBSP5—bread with 5% broccoli stalk powder addition; BBSP10—bread with 10% broccoli stalk powder addition; BBIP3—bread with 3% broccoli inflorescence powder addition; BBIP5—bread with 5% broccoli inflorescence powder addition; BBIP10—bread with 10% broccoli inflorescence powder addition. GA: General aspect; CTS: Crumb texture and structure; CT: Crust thickness; CsC: Crust color; E: Elasticity; Sp: Spreadability: F: Flexibility; CmC: Crumb color; CA: Crumb aeration; CHS: Crumb humidity/stickiness; CV: Crumb volume; Su: Sulphur; F: Freshness. Within each column, different letters are associated with significant differences according to Duncan’s test at *p* ≤ 0.05.

**Table 9:** Statistically significant differences between the experimental treatments in terms of the sensory features of bread added with broccoli plant part powder.

ET	Cr	N	Y	Ce	M	PN	G	Ca	Bu	FB	Bi	Sa	Sw
UC	0.86 ± 0.34 d	2.43 ± 2.94 de	2.00 ± 1.62 d	5.57 ± 2.97 a	1.00 ± 0.23 d	1.14 ± 0.86 c	1.43 ± 1.50 d	1.43 ± 0.50 d	1.57 ± 3.15 bc	8.29 ± 2.00 a	1.00 ± 0.25 c	4.86 ± 3.18 a	4.43 ± 3.15 a
BBSP3	6.29 ± 2.42 c	1.86 ± 1.10 e	2.43 ± 2.18 cd	4.00 ± 3.98 bc	1.86 ± 0.83 cd	6.00 ± 2.68 b	5.57 ± 2.87 c	1.71 ± 0.99 cd	1.86 ± 3.27 ab	7.29 ± 2.78 ab	1.57 ± 0.90 c	4.14 ± 3.08 b	3.29 ± 3.27 ab
BBSP5	6.71 ± 2.72 bc	3.57 ± 2.55 bc	2.71 ± 2.09 c	3.57 ± 2.98 cd	3.43 ± 3.17 b	8.29 ± 1.57 a	6.86 ± 2.93 ac	1.86 ± 1.10 bc	2.00 ± 3.02 a	7.29 ± 2.84 ab	3.00 ± 2.64 b	4.00 ± 2.96 bc	2.57 ± 3.02 ab
BBSP10	8.43 ± 2.39 ab	3.00 ± 3.17 cd	2.86 ± 3.13 bc	3.14 ± 2.67 cd	4.14 ± 3.11 ab	8.71 ± 1.30 a	8.00 ± 1.99 a	2.00 ± 1.30 bc	2.00 ± 0.71 a	6.57 ± 3.17 bc	4.43 ± 3.25 a	3.43 ± 2.59 cd	1.43 ± 0.71 b
BBIP3	7.00 ± 2.71 bc	3.29 ± 2.83 bc	2.86 ± 2.62 bc	4.57 ± 3.80 b	3.00 ± 2.23 bc	8.00 ± 2.28 a	6.00 ± 3.18 bc	1.71 ± 1.02 cd	1.43 ± 0.36 c	6.86 ± 2.59 ab	4.43 ± 3.34 a	3.14 ± 2.34 de	1.29 ± 0.46 b
BBIP5	8.14 ± 2.43 ab	3.86 ± 3.35 b	3.43 ± 3.50 ab	3.57 ± 2.93 cd	3.57 ± 2.77 ab	8.57 ± 1.74 a	6.29 ± 3.26 ac	2.14 ± 1.35 ab	1.57 ± 0.36 bc	6.00 ± 2.60 bc	4.57 ± 3.59 a	3.00 ± 2.33 de	1.14 ± 0.36 b
BBIP10	9.71 ± 0.46 a	4.71 ± 3.68 a	3.57 ± 3.46 a	3.00 ± 2.28 d	4.71 ± 3.62 a	9.00 ± 1.36 a	7.43 ± 3.00 ab	2.43 ± 2.00 a	1.86 ± 1.90 ab	5.14 ± 2.97 c	4.86 ± 3.82 a	2.71 ± 1.76 e	1.14 ± 0.36 b

ET: Experimental treatment; UC: untreated control; BBSP3—bread with 3% broccoli stalk powder addition; BBSP5—bread with 5% broccoli stalk powder addition; BBSP10—bread with 10% broccoli stalk powder addition; BBIP3—bread with 3% broccoli inflorescence powder addition; BBIP5—bread with 5% broccoli inflorescence powder addition; BBIP10—bread with 10% broccoli inflorescence powder addition. Cr: Cruciferous; N: Nut; Y: Yeast; Ce: Cereal; M: Mineral; PN: Plant notes; G: Grassy; Ca: Caramelized; Bu: Burned; FB: Fresh bread; Bi: Bitter; Sa: Salt; Sw: Sweet. Within each column, different letters are associated with significant differences according to Duncan’s test at *p* ≤ 0.05.

As can be observed in Figure 7, the control sample exhibited the highest mean scores for crumb volume, aeration and perceived moisture, confirming the optimal gluten network development and efficient gas retention during fermentation and baking. The progressive reduction in the mentioned parameters with the rising broccoli powder addition suggests a weakening effect on the gluten network and an interference of plant fiber with the alveolar structure formation [9].

Broccoli powder incorporation evidently increased the color intensity of bread crust and crumb, more pronounced upon the addition with 5% and 10% broccoli inflorescence powder, presumably due to the high content of natural pigments (chlorophylls, carotenoids, and phenolic compounds) present in broccoli florets, altering the product color appearance and promoting browning reactions during baking.

The bread added with 3% of broccoli stalk and inflorescence powder showed similar scores to the untreated control for crumb structure and elasticity. However, at 10% concentration, for both the stalks and inflorescences, a decrease in crumb elasticity and aeration was recorded, accompanied by structural density augmentation, caused by the high water absorption capacity of insoluble fibers and by the disruption of the gluten matrix continuity, which limits the expansion of gas cells during proofing [28].

Broccoli inflorescence powder elicited more sensory changes, compared to the stalks, especially regarding the color and crumb characteristics, which can relate to higher phenolic, pigment and dietary fiber level. However, the bread with 3 and 5% broccoli inflorescence powder addition provides the best compromise between functional values and sensorial acceptability, i.e., a favorable balance between visual aspect, crumb consistency and mechanical properties.

The control samples exhibited the highest intensity of typical bakery-related descriptors, particularly fresh bread, cereal, and yeast notes (Figure 8), which contribute to the traditional sensory identity of wheat bread and were progressively altered following broccoli powder incorporation.

Increase in cruciferous, freshness, plant notes, and grassy descriptors was recorded with the rising broccoli powder addition, especially at 5 and 10% inflorescence concentrations, indicating that the latter plant part contributed more strongly to the volatile profile than stalk powder. The mentioned behavior is presumably due to the higher levels of glucosinolates, sulfur-containing compounds, chlorophylls, and phenolic constituents naturally present in broccoli florets; particularly, during dough fermentation and baking, the degradation products of glucosinolates, including isothiocyanates and sulfur-containing volatiles, may contribute significantly to the characteristic cruciferous aroma [31]. An increase in the mentioned sensory characteristics was reported in a case of broccoli powder integration into crackers by up to 12.5% [28], compared to the untreated control.

An augmentation in sulfur-related notes was also detected upon broccoli powder addition, which was more evident with the 10% inflorescence powder integration, reflecting the naturally high sulfur compound content in *Brassica* plants. In this respect, sulfur-containing volatiles are recognized contributors to broccoli aroma and may positively influence the perceived product authenticity at moderate concentrations, while excessive intensities could negatively affect consumer acceptance.

Interestingly, the incorporation of broccoli powder resulted in a gradual reduction in yeast, cereal, and fresh bread descriptors, particularly at higher addition levels, suggesting that broccoli-derived volatiles partially masked traditional bakery aromas or altered the balance of aroma-active compounds generated during fermentation and baking. Similar masking effects were reported in fiber-enriched bread, where plant ingredients compete with cereal-derived aroma compounds for sensory dominance [43].

The caramelized and burned notes remained relatively low in all formulations, indicating that broccoli addition did not substantially modify thermal reaction pathways responsible for these aroma descriptors; additionally, sweet and salted perceptions showed small variations between the experimental treatments.

Regarding the overall sensory acceptance, broccoli-derived ingredients significantly reshaped the aromatic profile of bread, and moderate inclusion levels (3–5%) provided the most favorable balance between characteristic bread aroma and freshness and distinctive plant notes associated with broccoli bioactive compounds. Conversely, the highest broccoli incorporation level (10%), particularly as inflorescence powder, generated more intense cruciferous, grassy, and sulfurous attributes, beyond which consumer preference may decline despite improved functional and nutritional value.

The mentioned findings support the potential application of broccoli plant parts and waste as functional bakery ingredients capable of enhancing sensory complexity while contributing additional health-promoting phytochemicals.

In previous research [4], bread with added broccoli powder had pronounced broccoli aroma and flavor. Moreover, the incorporation of 5% grape pomace improved the nutritional profile of bread without compromising consumer acceptability [33]. In another study [27], the most appreciable nutritional value and sensory characteristics were recorded in cakes fortified with 4% grape powder and the best texture upon 6% integration. In the latter investigations, an over 10% grape pomace incorporation caused color darkening and, accordingly, product appearance worsening.

## 4. Conclusions

From this study, it arose that the increasing integration percentage from 3 to 10% of *Brassica oleracea* var. italica plant parts to fortify ‘White Pan Bread’, enhanced quality and antioxidant properties, despite having the opposite effect on textural and porosity properties. The lowest additions of stalk and inflorescence powder to bread provided the best compromise between structural characteristics and aromatic profile, compared to the control, showing cruciferous plant aromatic notes which differentiate the product from the conventional one. Contrastingly, the 10% broccoli powder integration led to the intensification of plant and sulfur attributes, associated with crumb volume and aeration reduction, i.e., technological and sensory limitations.

This research allowed us to identify interesting perspectives to create novel functional food from ‘White Pan Bread’ production by integrating wheat flour with broccoli plant parts as functional ingredients, aiming to enhance the food’s nutritional value without compromising sensory quality under a sustainable strategy.

Future research should deepen the investigation regarding the consumer acceptability of bread with added broccoli plant part powder, under the current conditions of the bakery industry.

## Figures and Tables

**Figure 1:**
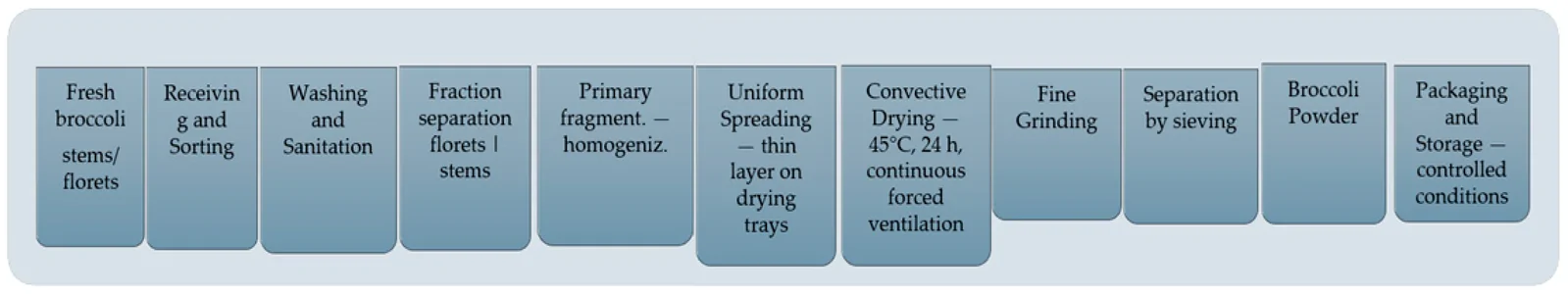
Technological process for drying broccoli to produce powder.

**Figure 2:**
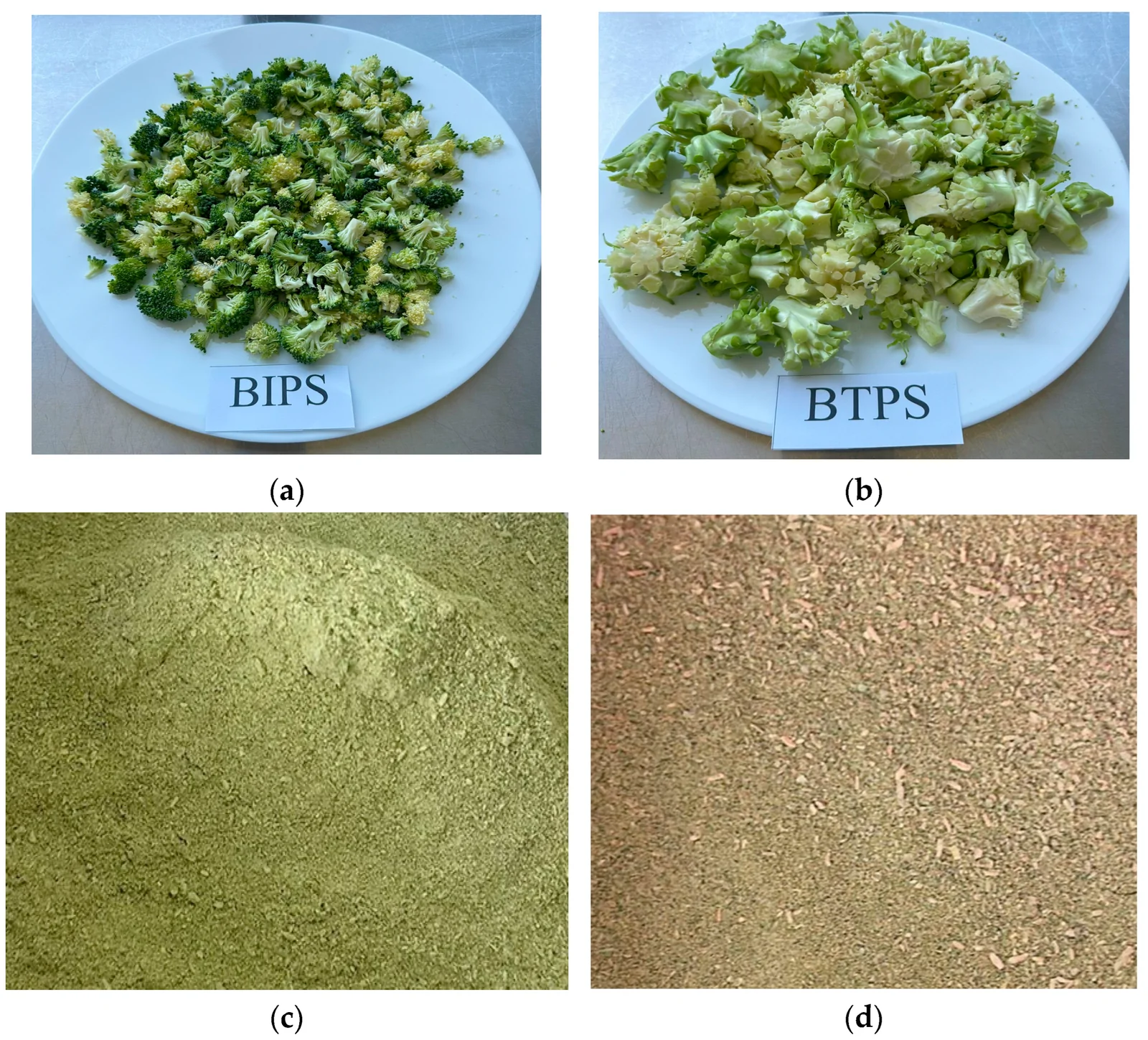
(**a**) Broccoli inflorescences chopped; (**b**) broccoli stalks chopped; (**c**) broccoli inflorescence powder; (**d**) broccoli stalk powder.

**Figure 3:**
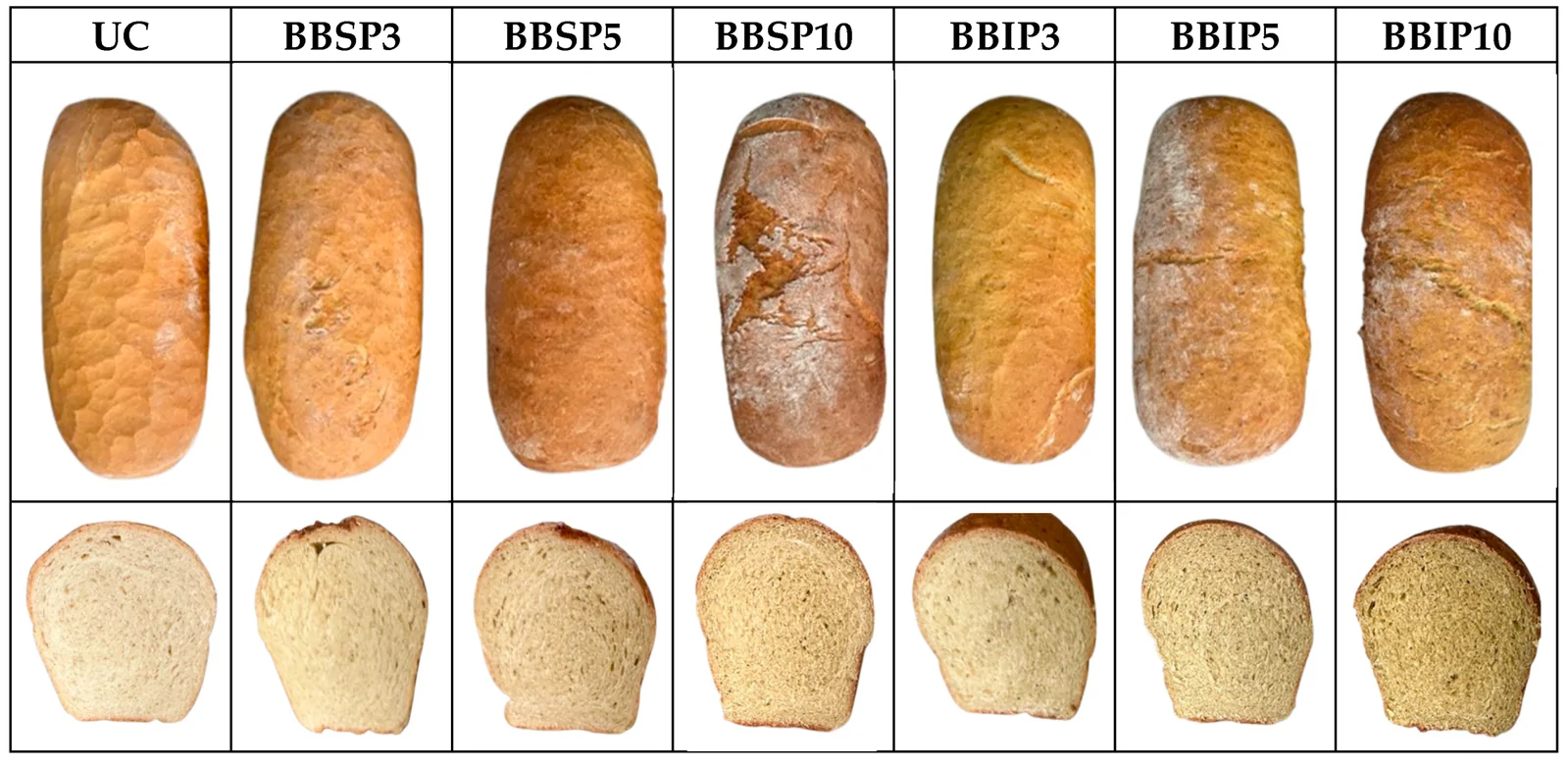
The aspect of the developed bread samples. UC—untreated control; BBSP3—bread with 3% broccoli stalk powder addition; BBSP5—bread with 5% broccoli stalk powder addition; BBSP10—bread with 10% broccoli stalk powder addition; BBIP3—bread with 3% broccoli inflorescence powder addition; BBIP5—bread with 5% broccoli inflorescence powder addition; BBIP10—bread with 10% broccoli inflorescence powder addition.

**Figure 4:**
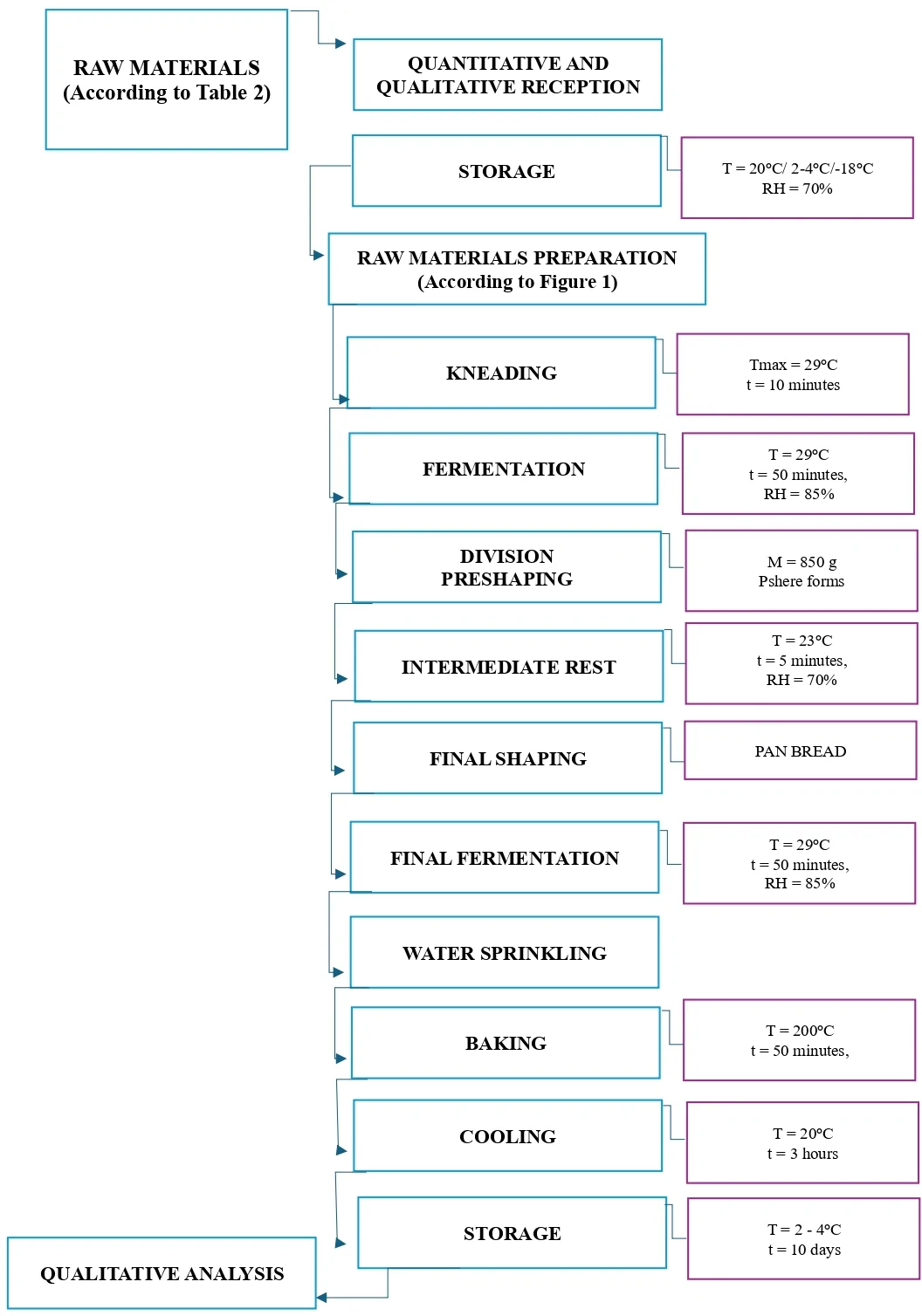
Technological scheme for functional bread preparation.

**Figure 5:**
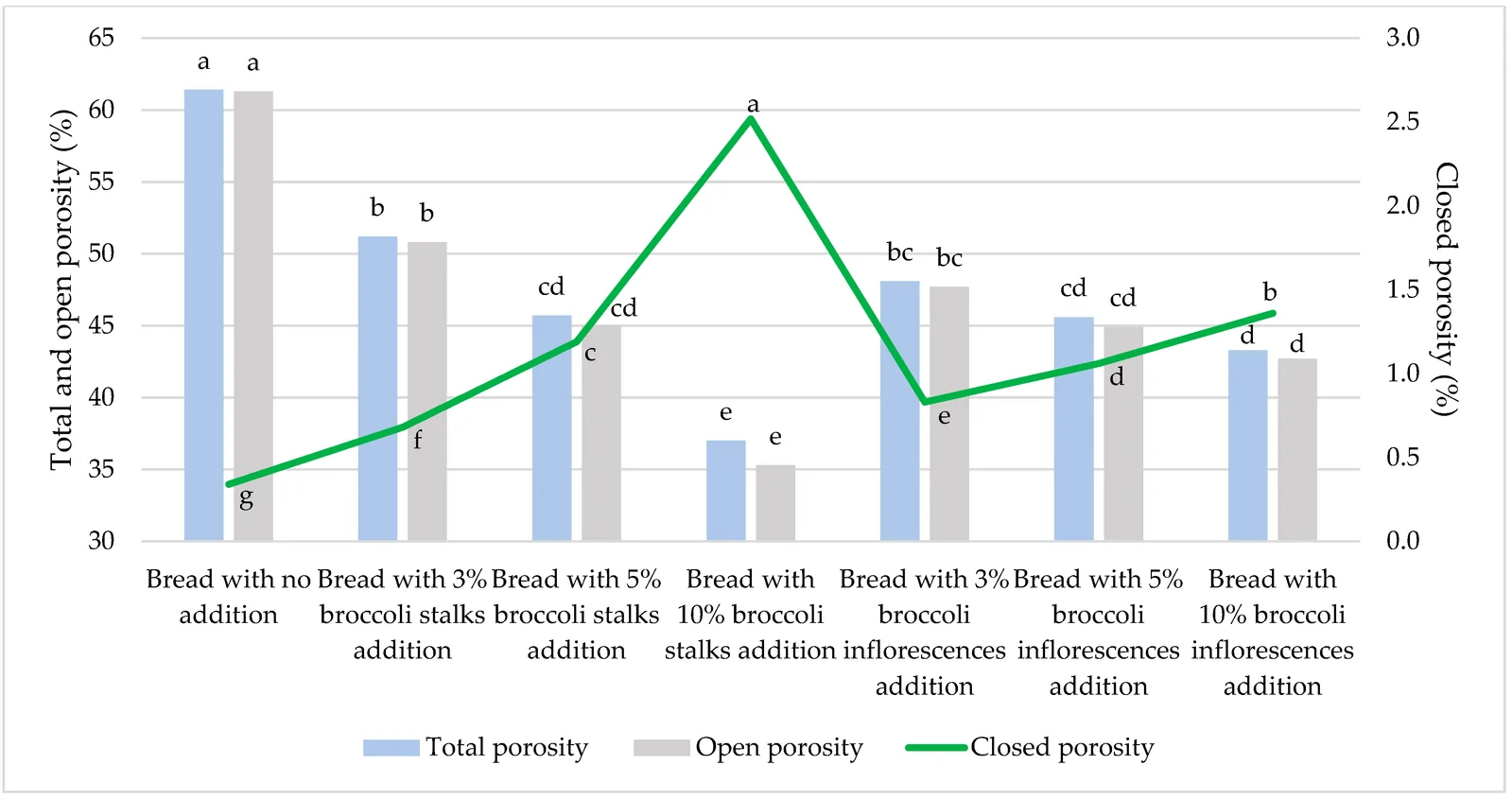
Effect of addition percentage of broccoli stalk and inflorescence powder on bread porosity. Different letters are associated with significant differences according to Duncan’s test at *p* ≤ 0.05.

**Figure 6:**
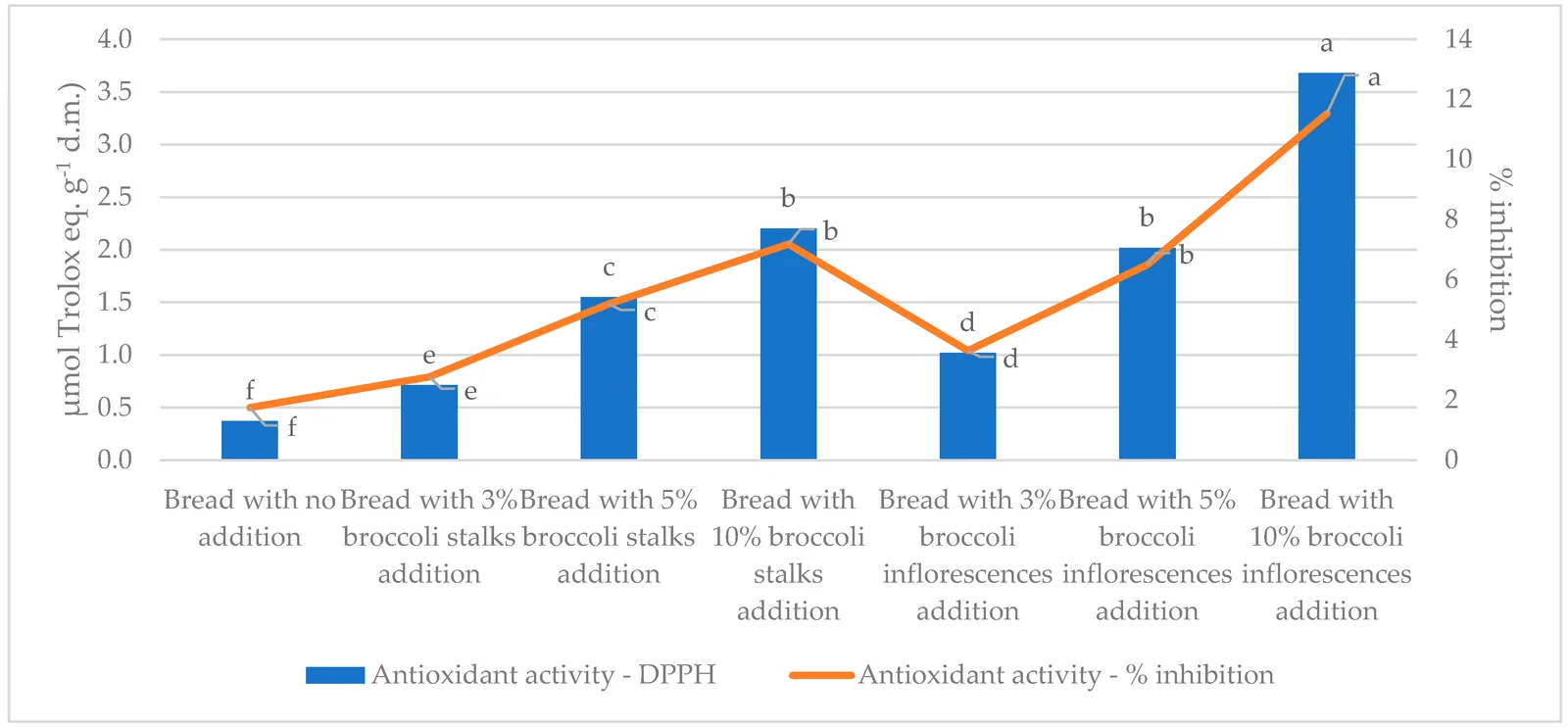
Effect of addition percentage of broccoli stalk and inflorescence powder on bread antioxidant activities. Different letters are associated with significant differences according to Duncan’s test at *p* ≤ 0.05.

**Figure 7:**
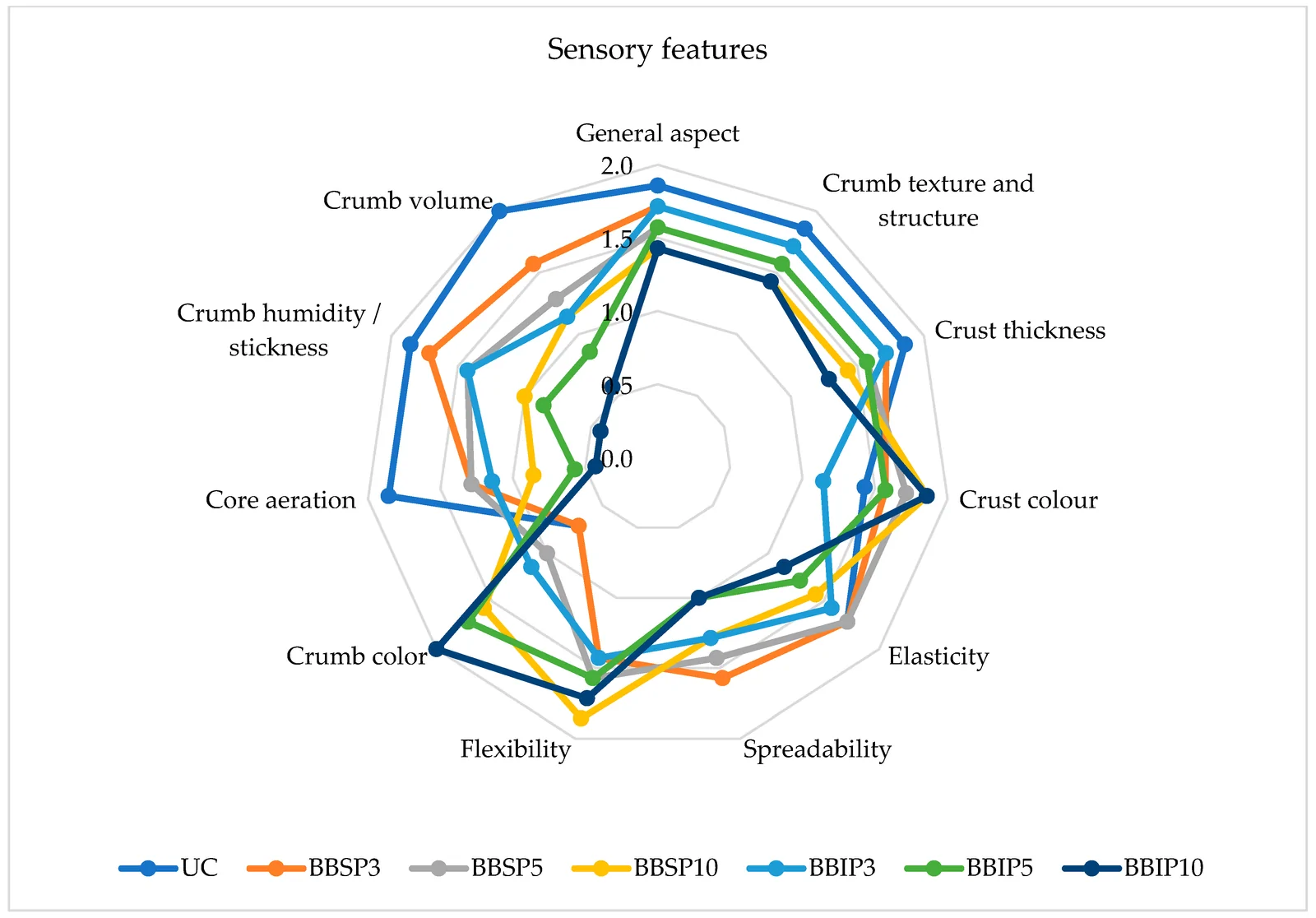
Sensory features of bread added with broccoli stalk and inflorescence powder. UC—untreated control; BBSP3—bread with 3% broccoli stalk powder addition; BBSP5—bread with 5% broccoli stalk powder addition; BBSP10—bread with 10% broccoli stalk powder addition; BBIP3—bread with 3% broccoli inflorescence powder addition; BBIP5—bread with 5% broccoli inflorescence powder addition; BBIP10—bread with 10% broccoli inflorescence powder addition.

**Figure 8:**
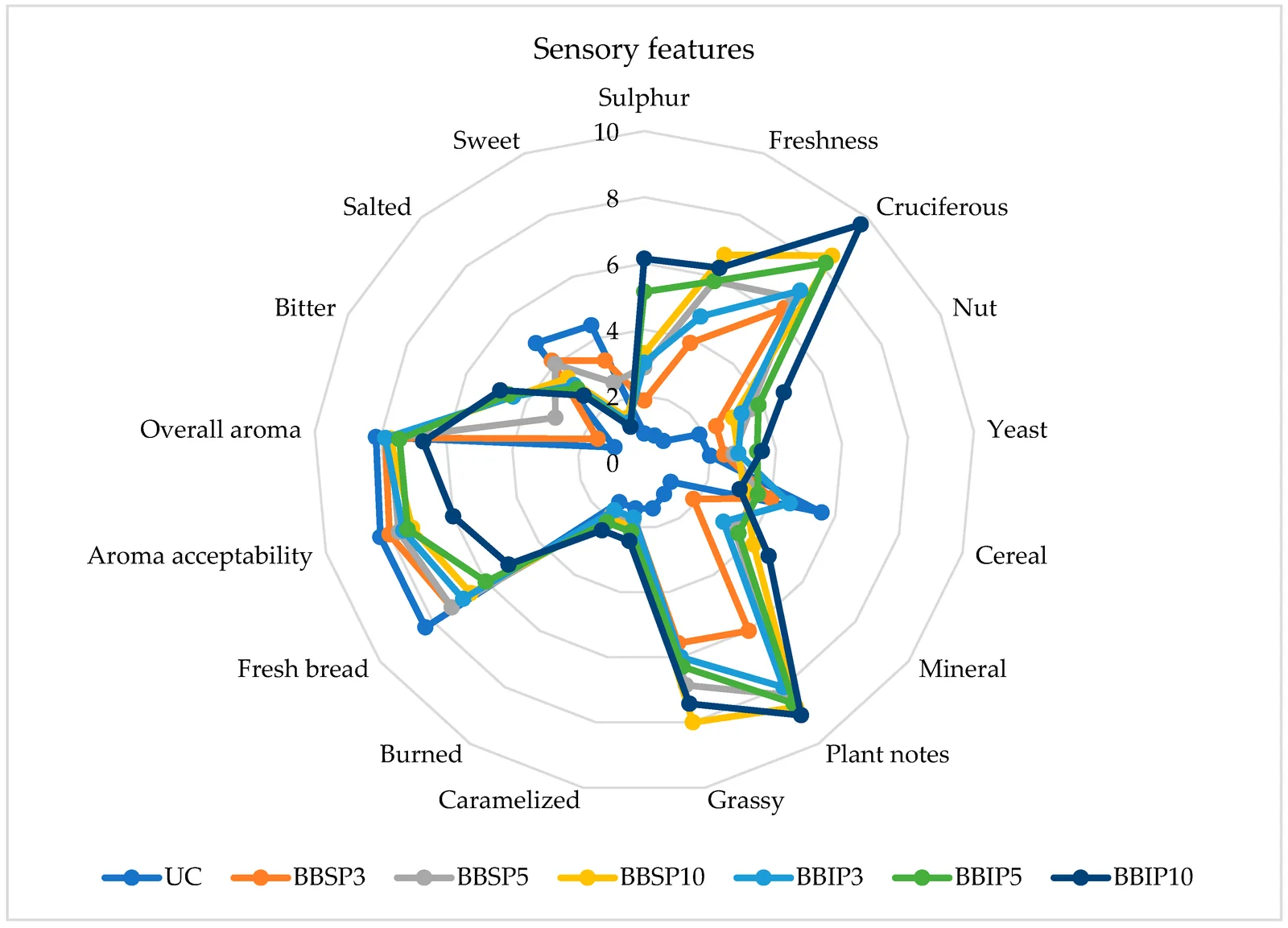
Sensory features of bread added with broccoli stalk and inflorescence powder. UC—untreated control; BBSP3—bread with 3% broccoli stalk powder addition; BBSP5—bread with 5% broccoli stalk powder addition; BBSP10—bread with 10% broccoli stalk powder addition; BBIP3—bread with 3% broccoli inflorescence powder addition; BBIP5—bread with 5% broccoli inflorescence powder addition; BBIP10—bread with 10% broccoli inflorescence powder addition.

**Table 1:** Chemical composition of broccoli stalk and inflorescence powder integrated into bread.

Broccoli Powder	Dry Matter	Sugars	Fiber	Proteins	Fats	Ash	Vitamin C	Pol	Fl	TC
	g 100 g^−1^	g 100 g^−1^					mg 100 g^−1^	mg g^−1^		
Stalks	85.3 ± 2.5	12.2 ± 0.4	52.5 ± 1.4	15.7 ± 0.6	6.9 ± 0.2	10.8 ± 0.3	150 ± 5.6	9.7 ± 0.3	6.0 ± 0.2	0.17 ± 0.01
Inflorescences	86.8 ± 2.7	13.4 ± 0.4	42.8 ± 1.2	28.6 ± 1.0	5.0 ± 0.2	8.3 ± 0.3	260 ± 9.2	11.4 ± 0.4	5.3 ± 0.2	0.19 ± 0.01

Pol: Polyphenols; Fl: Flavonoids; TC: Total carotenoids. The values are expressed on a dry weight basis.

**Table 2:** The recipes of the untreated control and broccoli-enriched bread.

Ingredients	UC	BBSP3	BBSP5	BBSP10	BBIP3	BBIP5	BBIP10
White flour (%)	59.8	58.6	57.3	53.3	58.6	57.3	53.3
Water (%)	35.8	35.2	35.5	37.0	35.2	35.5	37.0
Yeast (%)	2.9	2.9	2.9	2.9	2.9	2.9	2.9
Broccoli powder (%)	-	1.8	2.8	5.3	1.8	2.8	5.3
Salt (%)	0.9	0.9	0.9	0.9	0.9	0.9	0.9
Sugar (%)	0.6	0.6	0.6	0.6	0.6	0.6	0.6

UC—untreated control; BBSP3—bread with 3% broccoli stalk powder addition; BBSP5—bread with 5% broccoli stalk powder addition; BBSP10—bread with 10% broccoli stalk powder addition; BBIP3—bread with 3% broccoli inflorescence powder addition; BBIP5—bread with 5% broccoli inflorescence powder addition; BBIP10—bread with 10% broccoli inflorescence powder addition.

**Table 3:** Textural parameters of dough as affected by broccoli plant part and addition percentage.

Treatment	Flat Spheric Dough—Bidimensional Elasticity			Flat Strips Dough—Unidimensional Elasticity		
	MBS (N)	MDDS (mm)	MW (mJ)	MBS (N)	MDDS (mm)	MW (mJ)
UC	3.28 ± 0.18 a	42.4 ± 1.8 a	83.7 ± 2.2 a	1.80 ± 0.03 a	18.9 ± 1.4 a	17.2 ± 1.1 a
BBSP3	2.95 ± 0.13 ab	38.0 ± 2.3 b	70.8 ± 1.3 b	1.07 ± 0.06 b	16.9 ± 1.8 ab	14.2 ± 1.0 b
BBSP5	2.65 ± 0.23 bc	35.4 ± 2.2 bc	61.7 ± 2.3 c	0.70 ± 0.08 c	15.6 ± 1.3 bc	12.4 ± 0.8 c
BBSP10	1.60 ± 0.20 e	27.0 ± 2.3 d	39.4 ± 2.2 e	0.48 ± 0.13 d	14.9 ± 1.5 bc	8.2 ± 0.5 d
BBIP3	2.45 ± 0.23 cd	38.0 ± 1.9 b	61.7 ± 2.4 c	0.95 ± 0.04 b	17.8 ± 1.6 ab	14.2 ± 0.8 b
BBIP5	2.15 ± 0.18 d	33.9 ± 1.6 c	55.4 ± 2.2 d	0.71 ± 0.04 c	17.1 ± 1.5 ac	12.1 ± 0.8 c
BBIP10	1.30 ± 0.10 e	27.8 ± 1.7 d	30.1 ± 2.6 f	0.55 ± 0.05 d	14.5 ± 0.7 c	7.1 ± 0.8 d

UC—untreated control; BBSP3—bread with 3% broccoli stalk powder addition; BBSP5—bread with 5% broccoli stalk powder addition; BBSP10—bread with 10% broccoli stalk powder addition; BBIP3—bread with 3% broccoli inflorescence powder addition; BBIP5—bread with 5% broccoli inflorescence powder addition; BBIP10—bread with 10% broccoli inflorescence powder addition. MBS—Maximum breaking strength; MDDS—maximum deformation of dough sheet; MW—mechanical work. Within each column, values followed by different letters are significantly different at *p* ≤ 0.05 according to Duncan’s test.

**Table 4:** Textural properties of bread as affected by broccoli plant part and addition percentage.

Treatment	Firmness (N)	Consistency (mJ)	Cohesiveness	Elasticity	Resilience	Gumminess (N)	Chewiness (N)
UC	18.5 ± 0.8 d	129.9 ± 5.8 e	0.37 ± 0.02 c	0.99 ± 0.05 a	0.29 ± 0.02 a	7.3 ± 0.4 c	7.2 ± 0.3 e
BBSP3	33.3 ± 1.7 c	212.1 ± 9.3 d	0.38 ± 0.02 bc	0.86 ± 0.04 bc	0.13 ± 0.01 b	13.1 ± 0.6 b	9.7 ± 0.5 d
BBSP5	34.5 ± 1.8 c	223.9 ± 11.1 d	0.39 ± 0.03 bc	0.83 ± 0.05 bc	0.12 ± 0.01 bc	13.3 ± 0.4 b	10.8 ± 0.6 cd
BBSP10	36.5 ± 1.7 c	248.1 ± 12.1 c	0.42 ± 0.03 ab	0.79 ± 0.04 c	0.11 ± 0.01 bc	13.8 ± 0.6 b	11.9 ± 0.7 c
BBIP3	39.8 ± 1.9 b	261.5 ± 12.5 c	0.40 ± 0.02 ac	0.86 ± 0.04 bc	0.11 ± 0.01 bc	16.7 ± 0.9 a	14.5 ± 0.8 b
BBIP5	42.3 ± 2.0 b	285.4 ± 12.4 b	0.42 ± 0.02 ab	0.84 ± 0.05 bc	0.11 ± 0.01 bc	17.2 ± 0.9 a	15.0 ± 0.9 b
BBIP10	46.9 ± 2.1 a	320.2 ± 15.0 a	0.44 ± 0.03 a	0.81 ± 0.05 bc	0.10 ± 0.01 c	17.8 ± 0.9 a	16.9 ± 0.9 a

UC—untreated control; BBSP3—bread with 3% broccoli stalk powder addition; BBSP5—bread with 5% broccoli stalk powder addition; BBSP10—bread with 10% broccoli stalk powder addition; BBIP3—bread with 3% broccoli inflorescence powder addition; BBIP5—bread with 5% broccoli inflorescence powder addition; BBIP10—bread with 10% broccoli inflorescence powder addition. Within each column, values followed by different letters are significantly different at *p* ≤ 0.05 according to Duncan’s test.

**Table 5:** Quality parameters of bread as affected by broccoli plant part and addition percentage.

Treatment	Dry Matter (%)	Acidity (%)	pH	Proteins (%)	Lipids (%)	Fiber (%)	M.S. (%)	NaCl (%)	AW
UC	68.9 ± 1.0 a	1.47 ± 0.12 d	5.49 ± 0.02 e	7.66 ± 0.19 a	0.52 ± 0.05 a	1.9 ± 0.2 e	1.38 ± 0.05 e	0.85 ± 0.16	0.85 ± 0.01 a
BBSP3	67.2 ± 1.0 ab	1.87 ± 0.12 cd	5.56 ± 0.02 d	7.54 ± 0.17 ab	0.43 ± 0.04 bc	3.4 ± 0.2 c	1.73 ± 0.07 bc	0.83 ± 0.20	0.83 ± 0.01 bc
BBSP5	65.9 ± 0.5 bc	1.93 ± 0.06 cd	5.59 ± 0.01 cd	7.45 ± 0.09 ac	0.40 ± 0.03 c	5.2 ± 0.7 b	1.82 ± 0.03 b	0.83 ± 0.20	0.83 ± 0.01 bc
BBSP10	64.1 ± 1.0 cd	3.17 ± 0.15 b	5.65 ± 0.02 b	7.19 ± 0.12 cd	0.36 ± 0.03 cd	7.3 ± 0.7 a	2.06 ± 0.09 a	0.82 ± 0.22	0.82 ± 0.01 c
BBIP3	64.5 ± 1.2 cd	2.37 ± 0.21 c	5.57 ± 0.01 d	7.30 ± 0.17 bd	0.49 ± 0.05 ab	2.7 ± 0.3 d	1.44 ± 0.03 de	0.83 ± 0.20	0.84 ± 0.01 ab
BBIP5	62.9 ± 1.3 d	2.40 ± 0.20 c	5.61 ± 0.03 c	7.04 ± 0.22 d	0.41 ± 0.03 c	3.7 ± 0.2 c	1.54 ± 0.04 d	0.83 ± 0.20	0.84 ± 0.01 ab
BBIP10	60.7 ± 1.2 e	4.13 ± 0.25 a	5.69 ± 0.03 a	6.72 ± 0.15 e	0.32 ± 0.04 d	5.6 ± 0.3 b	1.70 ± 0.05 c	0.82 ± 0.20	0.83 ± 0.01 bc
								n.s.	

UC—untreated control; BBSP3—bread with 3% broccoli stalk powder addition; BBSP5—bread with 5% broccoli stalk powder addition; BBSP10—bread with 10% broccoli stalk powder addition; BBIP3—bread with 3% broccoli inflorescence powder addition; BBIP5—bread with 5% broccoli inflorescence powder addition; BBIP10—bread with 10% broccoli inflorescence powder addition. M.S.: Mineral substances; AW: water activity. The values are expressed on fresh weight basis. n.s.: not significant; within each column, values followed by different letters are significantly different at *p* ≤ 0.05 according to Duncan’s test.

**Table 6:** Color components of dough and bread added with different percentages of broccoli stalk and inflorescence powder.

Treatment	Color Component								
	L			a*			b*		
	Dough	Bread Crust	Bread Crumb	Dough	Bread Crust	Bread Crumb	Dough	Crust	Crumb
UC	85.1 ± 0.6 a	63.3 ± 0.9 a	78.2 ± 0.6 a	0.25 ± 0.01 a	13.2 ± 0.5 a	−2.06 ± 0.02 f	21.5 ± 0.5 e	34.3 ± 1.3 a	22.5 ± 0.4 f
BBSP3	80.6 ± 0.4 b	58.8 ± 1.7 b	74.3 ± 0.5 b	−4.08 ± 0.11 b	12.1 ± 0.7 ac	−0.84 ± 0.08 b	25.9 ± 0.2 d	27.9 ± 1.5 bc	24.8 ± 0.3 e
BBSP5	79.5 ± 0.5 c	57.6 ± 0.8 b	70.5 ± 0.5 c	−5.51 ± 0.08 c	12.0 ± 1.2 ac	−0.77 ± 0.03 b	27.1 ± 0.2 c	24.7 ± 2.0 cd	26.7 ± 0.3 c
BBSP10	74.2 ± 0.7 e	53.9 ± 0.3 c	67.7 ± 0.6 d	−7.16 ± 0.10 d	11.3 ± 0.5 c	−0.27 ± 0.04 a	29.2 ± 0.3 b	22.9 ± 2.0 d	28.3 ± 0.3 b
BBIP3	76.3 ± 0.9 d	54.4 ± 0.3 c	70.4 ± 0.5 c	−7.30 ± 0.15 d	12.9 ± 0.2 ab	−1.94 ± 0.05 e	26.9 ± 0.7 c	30.2 ± 2.0 b	25.8 ± 0.3 d
BBIP5	71.7 ± 1.0 f	53.5 ± 1.5 c	67.4 ± 0.2 d	−9.00 ± 0.05 e	11.7 ± 0.5 bc	−1.68 ± 0.07 d	29.5 ± 0.2 b	28.5 ± 1.0 bc	27.9 ± 0.4 b
BBIP10	65.4 ± 0.2 g	46.8 ± 1.6 d	60.3 ± 0.1 e	−10.19 ± 0.13 f	11.4 ± 0.5 c	-0.99 ± 0.09 c	30.4 ± 0.2 a	25.5 ± 1.8 cd	29.4 ± 0.4 a

UC—untreated control; BBSP3—bread with 3% broccoli stalk powder addition; BBSP5—bread with 5% broccoli stalk powder addition; BBSP10—bread with 10% broccoli stalk powder addition; BBIP3—bread with 3% broccoli inflorescence powder addition; BBIP5—bread with 5% broccoli inflorescence powder addition; BBIP10—bread with 10% broccoli inflorescence powder addition. Within each column, values followed by different letters are significantly different at *p* ≤ 0.05 according to Duncan’s test.

**Table 7:** Antioxidant parameters of bread as affected by broccoli plant part and addition percentage.

Treatment	Vitamin C (mg 100 g^−1^)	TP (mg GAE g^−1^)	Flavonoids (mg CE g^−1^)	TC (mg g^−1^)	β-Carotene (mg g^−1^)	Licopene (mg g^−1^)
UC	0.0 ± 0.0 e	0.10 ± 0.04 f	0.10 ± 0.01 e	0.000 ± 0.000 e	0.000 ± 0.000 b	0.000 ± 0.000 d
BBSP3	3.2 ± 0.3 d	0.20 ± 0.02 e	0.16 ± 0.02 d	0.065 ± 0.001 d	0.039 ± 0.002 a	0.003 ± 0.000 c
BBSP5	5.1 ± 0.5 c	0.34 ± 0.03 d	0.27 ± 0.01 c	0.070 ± 0.002 cd	0.041 ± 0.002 a	0.003 ± 0.000 c
BBSP10	10.6 ± 0.8 b	0.48 ± 0.04 b	0.37 ± 0.01 b	0.073 ± 0.002 bc	0.042 ± 0.004 a	0.004 ± 0.000 c
BBIP3	5.6 ± 1.4 c	0.23 ± 0.02 e	0.17 ± 0.01 d	0.070 ± 0.002 cd	0.041 ± 0.005 a	0.003 ± 0.000 b
BBIP5	9.6 ± 0.8 b	0.41 ± 0.02 c	0.35 ± 0.01 b	0.078 ± 0.002 ab	0.042 ± 0.004 a	0.004 ± 0.000 b
BBIP10	17.2 ± 1.0 a	0.73 ± 0.03 a	0.50 ± 0.03 a	0.084 ± 0.003 a	0.044 ± 0.002 a	0.005 ± 0.000 a

The values are expressed on a dry weight basis, except for vitamin C which is expressed on a fresh weigh basis. UC—untreated control; BBSP3—bread with 3% broccoli stalk powder addition; BBSP5—bread with 5% broccoli stalk powder addition; BBSP10—bread with 10% broccoli stalk powder addition; BBIP3—bread with 3% broccoli inflorescence powder addition; BBIP5—bread with 5% broccoli inflorescence powder addition; BBIP10—bread with 10% broccoli inflorescence powder addition; TP: total polyphenols; TC: total carotenoids. Within each column, values followed by different letters are significantly different at *p* ≤ 0.05 according to Duncan’s test.

## Data Availability

The original contributions presented in this study are included in the article. Further inquiries can be directed to the corresponding authors.
